# The Cross-Cultural Competencies and Attitudes Toward Ultraorthodox Clients Among Secular Therapists in Israel: An Explanatory Study

**DOI:** 10.3390/healthcare13101210

**Published:** 2025-05-21

**Authors:** Einat Doron, Dariusz Walkowiak, Rivka Tuval-Mashiach, Sławomir Tobis, Jan Domaradzki

**Affiliations:** 1 Independent Researcher, Binyamina 3052833, Israel; 2 Department of Organization and Management in Health Care, Poznan University of Medical Sciences, 60-356 Poznan, Poland; 3 Faculty of Social Sciences, Department of Psychology, Bar-Ilan University, Ramat Gan 5290002, Israel; 4 Department of Occupational Therapy, Poznan University of Medical Sciences, 60-608 Poznan, Poland; stobis@ump.edu.pl; 5 Department of Social Sciences and Humanities, Poznan University of Medical Sciences, 60-806 Poznan, Poland; jandomar@ump.edu.pl

**Keywords:** attitudes, cross-cultural competencies, Israel, Haredim, secular therapists, cross-cultural therapy, ultraorthodox Jews

## Abstract

Background: To provide effective mental health care across cultural differences, therapists must develop cultural competencies, including an awareness of and sensitivity to diverse backgrounds. In Israel, secular therapists (STs) working with ultra-orthodox (UO), also referred to as Haredi, clients face challenges due to mutual distrust and sociopolitical tensions between the communities. This study aims to assess Israeli STs’ cross-cultural competencies and attitudes toward Haredi clients, examining the association between perceptions and competencies. Methods: The data were collected from an anonymous, self-administered online survey conducted between April and November 2024 among 70 STs in Israel. The study utilized the *Cross-Cultural Competence of Healthcare Professionals (CCCHP-27)* scale and a self-developed questionnaire assessing STs’ attitudes toward UO clients. The statistical analysis was performed using JASP 0.18.3. Results: While STs exhibited high general cross-cultural competence, their competence in working with Haredi clients was lower and more complex. Therapists with more experience treating UO clients had more positive attitudes toward them, whereas those working in public settings demonstrated lower cultural awareness. Although STs faced cultural and ideological challenges, many expressed openness and professional growth. Notably, while 57.2% felt that working with UO clients improved their skills, only 37.2% recommended it to others. Similarly, while 52.8% enjoyed working with diverse clients, only 27.2% reported enjoying working with UO clients. A negative correlation was found between the *emotions about minorities* scale and two UO-related subscales, *views* (ρ = −0.307, *p* = 0.01) and *awareness* (ρ = −0.534, *p* < 0.001), suggesting that local sociopolitical factors influence attitudes toward the Haredi community. Conclusions: This study highlights a gap between STs’ general cross-cultural competencies and their attitudes toward the Haredi population. The findings underscore the need for continued professional training in culturally competent therapy, as personal interactions play a crucial role in bridging societal divides and improving therapeutic relationships.

## 1. Introduction

Due to globalization, many modern Western societies are characterized by linguistic, cultural, ethnic, and religious diversity. However, while ethno-cultural groups in multicultural societies engage with Western culture, their needs may be different, which makes intercultural communication unavoidable [1]. Consequently, health professionals should understand how culture and identity issues affect a person’s mental health. It is also important to recognize that Western societies themselves differ in the value systems and philosophical traditions they follow, which further complicates intercultural interactions not only between majority and minority groups, but also within the broader Western context itself, where competing ideologies and normative frameworks may shape perceptions of mental health, caregiving, and help-seeking behaviors. Thus, cross-cultural therapy has been discussed and researched for many years due to the changing nature of immigration around the globe in the past decades. Considering the intricate complexity and cultural variety that define modern societies, it is important to manage the tensions that arise from living in communities of diverse cultural origins [2].

This study builds on findings from a previous qualitative investigation that explored the experiences of secular therapists (STs) providing care to ultraorthodox (UO) clients in Israel [3]. That earlier research identified three major challenges: the therapists’ perceived outsider status, frequent axiological (value-based) conflicts during sessions, and the importance of establishing trust in the therapeutic relationship. These insights provided the theoretical and contextual foundation for the current quantitative analysis. Specifically, the present study explores assumptions derived from those findings, such as the relevance of professional experience and ideological distance in shaping therapists’ cultural competence and attitudes towards UO clients.

In addition, the previous study highlighted how therapeutic encounters are deeply embedded in Israel’s broader political and religious context. A climate of mutual suspicion prevails: many UO individuals view secular society as a threat to their values, while secular Israelis often regard the Haredim as socially and economically disengaged. This reciprocal distrust creates a complex cultural power dynamic in which therapists must navigate not only clinical challenges but also symbolic tensions rooted in wider societal divisions. As a result, the therapeutic relationship becomes both culturally and politically charged.

More and more therapists meet patients from different cultures, which necessitates changes in treatment to meet the various needs of multicultural populations. The cross-cultural encounter is a complex and dynamic meeting between personal values concerning world views originating in culture, beliefs, values, and emotional biases regarding different ethnic groups, as well as the identity and affiliation group of the therapist himself [4]. The ultimate aim, often called cultural competence, should be that members of minority groups are comfortable seeking help and the service providers are attuned to the nuances of individuals and their cultures [5]. This skill has been presented in numerous ways as a response to or remedy for the ethnocentric perspectives and practices that have historically shaped the Western intellectual sphere [2]. Cultural competency is often defined as encompassing two key components: cultural awareness and cultural sensitivity [6].

Cultural awareness involves gaining knowledge about diverse cultural groups by exploring multicultural content and recognizing the unique characteristics of each group. This process is primarily cognitive, focusing on the intellectual understanding and conscious processing of thoughts and actions as therapists become aware of them. Cultural sensitivity emphasizes the emotional aspect of cultural competency. It involves therapists responding to cultural differences with respect, care, and sensitivity. In this context, self-awareness and self-reflexivity emerge as crucial concepts [7]. Therapists are encouraged to recognize that their cultural history and personal experiences serve as a lens through which they view and interpret the experiences of clients from other cultural backgrounds. This understanding urges therapists to approach their work with a deeper awareness of their own biases and a greater openness to the cultural diversity of their clients [2].

Cross-cultural therapy must be understood not only as an interpersonal encounter but as a professional activity shaped by broader institutional, political, and ideological contexts. In societies marked by deep cultural and religious divides, such as Israel, the therapist–client relationship does not occur in a vacuum but is mediated by contested narratives about nationhood, modernity, and the legitimacy of moral authority. This model emphasizes how interconnected factors like racism, classism, sexism, and oppression contribute to understanding clients’ mental distress [8]. Psychotherapy happens in the interface between the social and the individual [9]. A culturally sensitive intervention, similar to Western approaches, aims to foster behaviors that enhance the patient’s quality of life [10], i.e., an individual’s subjective evaluation of their well-being, functioning, and satisfaction across physical, emotional, and social domains, in alignment with their personal aspirations and cultural and environmental values [11]. However, this does not involve promoting self-satisfaction or increasing self-awareness by expanding personal choices. Instead, intervention strategies tailored to traditional societies focus on helping patients cultivate harmony with their environment. They emphasize avoiding confrontation and stress the social and emotional effects of the individual’s decisions on their families and relatives [9]. Striving for an integrative awareness regarding class, heteronormativity, sexism, and ethnicity/culture is a continuous and challenging journey. Practitioners who have reached a level of understanding and awareness are more likely to engage in ethical practice across different cultures [12].

Israel’s demographic patterns and trends are unique, reflecting the country’s complex political, cultural, social, religious, and philosophical identity. Ultraorthodox (UO) or Haredi Judaism is the fastest-growing Jewish religious group in the 21st century, constituting 14% of Israel’s Jewish population [13]. It holds a distinct position as a culturally protected minority [14]. Members of this subgroup are committed to separating themselves from outside society. Their isolation is expressed through religious norms, behavioral practices, perceptions, education, and external appearances [15]. They often have minimal secular education, commonly speak Hebrew only as a second language, and may hold theological beliefs that conflict with the fundamental principles of modern care. UO communities are led by a rabbinic figure who advises on significant life decisions, including healthcare, with this guidance often overriding medical or legal advice. Patients and their families typically seek rabbinic counsel before pursuing mental health treatment, often trying intra-communal interventions first [16].

Although there is a noticeable movement toward the secular Western system of cultural values and norms, which emphasize individuality, cultural diversity, and an acceptance of differences—principles foundational to psychotherapy [15,17]—as well as changes in the concepts and practice of psychotherapy itself [18,19,20], the Haredi society, though comprised of many sub-groups, continues to express significant suspicion, distrust, and even hostility toward the secular general public [21]. They fear interacting with the secular world, viewing immodesty, materialism, and immoral behavior as existential threats to their community, which values strict obedience [16]. These tensions shape institutional arrangements in healthcare, where policies increasingly recommend matching religious clients with in-group therapists. While such policies are meant to foster cultural sensitivity, they may paradoxically reinforce cultural segregation and limit opportunities for cross-cultural understanding and bridge-building. Thus, an analysis of therapists’ attitudes must be situated within the complex and sometimes contradictory logic of an institutional adaptation to cultural pluralism.

While earlier studies examining therapeutic encounters between Haredi clients and secular therapists (STs) focused primarily on exploring the subjective experiences, emotional responses, and ethical dilemmas faced by either STs or Haredi clients during therapy e.g., Ref. [3], this paper intends to assess Israeli STs’ cultural competencies and their attitudes toward the Haredim. The previous qualitative study explored individual perspectives and challenges, such as an outsider status, value conflicts, and trust. In contrast, the present study employs a structured, quantitative approach to measure the prevalence, variation, and interrelations of these themes—now operationalized as measurable constructs—for a broader and more systematic analysis. An assessment of the cultural competence of healthcare professionals involved in direct patient care, such as psychotherapists and psychologists, is important to determine individual strengths and weaknesses, leading to self-awareness [21,22]. Thus, this study aims to answer the following research questions: (1) What is the level of cross-cultural competencies among STs working with UO clients in Israel? (2) What attitudes toward the Haredim do the Israeli STs hold? (3) What is the relationship between therapists’ cultural competence and their attitudes toward UO clients?

## 2. Methods

### 2.1. Study Design

As part of a broader project on the experiences of STs working with UO clients in Israel [3,15], this study focuses on the assessment of the Israeli STs’ cross-cultural competencies and attitudes toward the UO community. The overall project was designed as a two-phase study: an initial qualitative stage aimed at identifying key therapeutic challenges, followed by the present quantitative phase intended to assess the generalizability and prevalence of those findings. The quantitative study was conceived as a follow-up to a previously conducted qualitative investigation and was designed to explore whether the insights drawn from that earlier research could be generalized to a larger population of therapists. The study was designed as an anonymized, self-administered, computer-assisted online survey to assess STs’ attitudes, skills, and experiences when working with a segregated minority such as the UO Jews [22].

### 2.2. Participants and Setting

In the Israeli context, secularism constitutes a distinct cultural and social identity, typically linked with liberal or progressive beliefs and a worldview that stands in contrast to the conservative values of the ultraorthodox population. Therefore, in this study, the term “secular therapists” refers to therapists who personally identify as secular, clearly differentiating themselves from other religious affiliations, such as traditional, national-religious, orthodox, or ultraorthodox. This self-declared identity was used as a criterion for inclusion in the study. STs working with Haredi clients in Israel, were targeted for recruitment. These professions, psychologists, art therapists and clinical social workers, comprise the three main professions of psychotherapy in Israel, in addition, the frequent use of art therapy among the therapists reflects broader cultural considerations in working with ultraorthodox (UO) clients [20,23,24,25]. Art therapy offers a non-verbal, symbolic modality that suits the cultural sensitivities of the UO community, where direct verbal expression—particularly regarding emotional or taboo subjects—may be discouraged. It is often seen as less intrusive and more acceptable, enabling emotional exploration without breaching the norms of modesty or stigma.

Both because there is no official registry of STs working with UO clients and because of the current political situation in Israel, the participants were approached through social media platforms using several Facebook pages and specialized WhatsApp groups catering to the needs of therapists. Additionally, the recruitment process was continued using a non-probability snowball sampling method, as all eligible STs were asked to disseminate information about the survey among other potential participants.

The criteria for inclusion were as follows: (1) being a secular therapist; (2) being Israeli Jewish; (3) having a minimum of three years of professional experience in providing therapy; (4) having worked with at least two clients from the UO community; (5) having experience in working with both the general population and the UO community; (6) agreeing to take part in the study; (7) giving written informed consent to participate before completing the survey; and (8) having access to electronic devices and the ability to participate in an online survey.

Traditional or former religious therapists were excluded, as they may hold different views and skills on working with UO from secular therapists. Five participants with only UO supervisees and not clients were excluded, as the dynamics of a supervisory relationship differ from those of a traditional client–therapist relationship. Overall, 70 therapists completed the questionnaire, and two were excluded for not answering the profession criteria.

### 2.3. Research Tool

The European Statistical System’s guidelines were followed in developing the questionnaire [26]. The questionnaire used in this study comprised 74 closed-ended, single-choice questions divided into three parts (Supplementary Material S1). The first section contained questions regarding STs’ demographic characteristics. The second section included a self-developed original questionnaire designed to assess STs’ knowledge of and attitudes toward the Haredim. While it was constructed from themes based on a literature review, it consisted of 32 original items on the STs’ opinions on cross-cultural therapy with UO clients, their attitudes toward the UO community, their personal experiences with treating Haredim, and STs’ opinions on the benefits and challenges of treating UO clients. All these questions were assessed on a Likert scale answer ranging from 1 (completely disagree) to 5 (completely agree), with any “I neither agree nor disagree” responses considered as the midpoint of the response scale. To ensure the reliability of the information provided in an electronic survey, all questions were written in plain, clear, and unambiguous language. Both sections were designed by a research team consisting of three experts: two art therapists and a medical sociologist.

A self-designed set of questions was included to allow for individual responses to be analyzed while also enabling broader generalizations regarding the functioning of cross-cultural therapy with UO clients. The set of 32 questions was treated as a scale, with Cronbach’s alpha calculated to evaluate internal consistency alongside exploratory factor analyses (EFAs). The scale demonstrated a relatively high level of internal consistency, with Cronbach’s alpha at α = 0.806 (95% CI: 0.732–0.863), which is notable for a tool developed ad hoc. Using 24 of the 32 items, two factors were identified: *Views* (13 items: 1, 4, 6, 12, 13, 17, 23, 24, 25, 26, 27, 28, 29; α = 0.790) and *Awareness* (11 items: 7, 8, 9, 10, 14, 15, 19, 20, 21, 31, 32; α = 0.859). These factors were subsequently analyzed both as distinct scales and in terms of individual responses to specific content.

The third section included a validated cross-cultural competency assessment tool designed to assess Cross-Cultural Competence of Healthcare Professionals (CCCHP-27), which was developed by Bernhard et al. [27]. This self-administered multidimensional tool consists of 32 items divided into five domains measuring different dimensions of cross-cultural competence among healthcare professionals: (1) *motivation/curiosity*—nine items, e.g., “I consider working in a cross-cultural team an enrichment”; (2) *attitudes*—four items, e.g., “People who migrate should adapt to the local society, not the other way around”; (3) *skills*—five items, e.g., “I consider the values of patients in relation to family, religion, etc., if they seem relevant for the treatment”; (4) *emotions/empathy*—five items, e.g., “I prefer treating patients from my own cultural background to those who seem foreign to me”; and (5) *knowledge/awareness*—four items, e.g., “The migration experience is a critical life event and can be accompanied by psychosocial stress and health burden.” It also includes five questions assessing the impact of social desirability on the responses. The response format was also a 5-point Likert scale, ranging from 1—completely disagree to 5—completely agree. Higher scores correspond to higher cross-cultural competence.

Since there is no Hebrew version of CCCHP-27, following approval to use the CCCHP-27 obtained from its author, its English version was adapted to fit the local Israeli cultural context, and then it was back-translated. Firstly, the principal investigator (ED), an Israeli Jewish-trained art therapist working with UO clients, performed the forward translation. Next, it was back-translated for assurance of correctness by an external specialist fluent in Hebrew and English. Then, it was reviewed by a panel of three research experts: two art therapists and a medical sociologist. Finally, the co-authors made some final edits. While the questionnaire was translated precisely and without translation equivalence, specific terminology was modified to make it more appropriate for the Israeli context and the purpose of the study. For example, since the study aimed to assess STs’ cross-cultural competencies and attitudes toward the Haredim, the term “immigrants” was translated to “clients from different cultural backgrounds”. It was then tested on three STs. Finally, a focus group discussion with the study team was carried out, which led to a reformulation of five items. The final version of the questionnaire was pre-tested on two professionals via a communication platform and was again re-evaluated by the study team.

The original CCCHP-27 measures cross-cultural competence using five scales, with scale values calculated as the means of the corresponding raw item scores. It also includes items to assess the influence of social desirability on the responses. Validation studies conducted in various countries have not consistently confirmed the structural framework proposed by the tool’s creators. The internal consistency of the CCCHP-27 and its subscales was evaluated using Cronbach’s alpha. The factorial validity of the original scales was initially examined using an EFA with varimax rotation and ordinary least squares (OLS) for a minimum residual solution due to the non-normal distribution of items. Further evaluation of the factor structure and the number of dimensions relied on optimal coordinates, parallel analysis, eigenvalues greater than 1, and factor loadings exceeding 0.30. The overall CCCHP-27 demonstrated good internal consistency (α = 0.823, 95% CI: 0.801–0.903). The 27 items also identified three subscales related to cross-cultural competence: *emotions* (six items: 9, 12, 13, 23, 27, 31; α = 0.752), *attitudes* (seven items: 1, 3, 8, 15, 19, 28, 32; α = 0.582), *and skills* (five items: 4, 16, 22, 24, 25; α = 0.694). While the internal consistency values for some subscales, particularly attitudes, were moderate, they exceeded those observed during the original validation, indicating a potential for improvement in item selection and scale design [28].

### 2.4. Ethical Issues

The design of the study followed the Declaration of Helsinki (revised in 2000) [29] and was approved by the Poznan University of Medical Sciences Bioethics Committee (KB-139/23). Before completing the survey, written online informed consent was obtained from all the study participants by checking the “I agree” or “I do not agree” box on the online form.

### 2.5. Data Collection

The survey was conducted over eight months, from April to November 2024. First, the principal investigator posted an invitation letter with the link to the online questionnaire on Facebook pages and WhatsApp groups for therapists and invited all its members to participate. While convenience sampling was used in this study, STs were also recruited via phone calls and emails that were sent to relevant professional workplaces. Before starting the survey, all respondents were instructed by the principal investigator on the study’s purpose and character and the anonymous, voluntary, and confidential nature of the survey. They were also told that they could quit the survey at any given moment without any repercussions. After all affiliated therapists who met the inclusion criteria were invited to participate in the survey, they provided written informed consent to participate, and the survey was completed using electronic devices (for example, PCs, tablets, or smartphones), which took approximately 12–20 min to complete. Follow-up letters were posted on the same pages in June and November 2024, and more phone and email attempts were made to locate participants. Due to the ongoing Israeli–Gaza war, recruitment was significantly hindered, as many eligible participants experienced heightened stress levels and were unable or unwilling to participate. After six months with a declining response rate, data collection was extended for two additional months. As no new responses were received during that time, the data collection phase was concluded.

### 2.6. Data Analysis

Descriptive statistics were used to summarize the data, including medians, means with 95% confidence intervals (CIs), and standard deviations. Other data, such as responses to Likert scale questions, are presented as frequencies and percentages of total responses. The Shapiro–Wilk test was applied to assess the normality of the data distribution, along with the Brown–Forsythe test to evaluate the homogeneity of variances. Comparisons between groups, categorized based on sociodemographic and professional characteristics, were conducted using Welch’s *t*-test for data meeting the required assumptions and the Mann–Whitney U test for non-parametric data.

## 3. Results

Table 1 shows the sociodemographic characteristics of the respondents. In total, 70 respondents completed the survey, of which 26 were psychologists (37.1%), 23 were art therapists (32.9%) and 21 were clinical social workers (30%), all of Israeli origin. There was a predominance of female respondents (87.1%); however, this disproportion results from the fact that therapeutic professions are strongly gendered in Israel [30]. While respondents’ professional experience ranged from 4–50 years (mean 18.3), their professional experience working with Haredi clients ranged from 2–44 years (mean 11.3), and the mean number of Haredi clients over the years was 29 (range 17–41).

Table 2 shows the STs’ answers to the general cross-cultural competency questionnaire (CCCHP-27). Responses were grouped into three categories: *emotions* (six items: 9, 12, 13, 23, 27, 31), *attitudes* (seven items: 1, 3, 8, 15, 19, 28, 32), and *skills* (five items: 4, 16, 22, 24, 25) (for the full results, see Supplementary Material S2, Table S1).

The scores on the *emotions* scale suggest that most STs in this study feel comfortable and open to working with culturally diverse clients, with positive emotions regarding this encounter. More than half of respondents (54.3%) have expressed disagreement with the statement that they prefer treating clients from their own cultural background rather than those who seem foreign. Similarly, 58.6% disagreed with the idea that professional interactions with clients from different cultural backgrounds often leave them feeling unsure, angry, or frustrated. Only 11.4% have agreed that they get impatient when they cannot make themselves understood by clients from different cultural backgrounds, and a small number (7.1%) found speaking slowly and using clear, straightforward language challenging with people who have difficulty understanding their instructions. Most (67.1%) have agreed that being part of a different cultural group is a critical life experience and can be accompanied by psychosocial stress and health burdens.

Scores on the *attitudes* scale reflect STs’ positive attitudes toward cultural diversity, their willingness to engage with different cultures, and their openness to learning and improving their cross-cultural competencies. A significant majority of participants (85.7%) emphasized the importance of treating clients in alignment with their cultural needs and individual values. Additionally, only 2.8% of STs disagreed with the notion that working with clients from different cultural backgrounds is an exciting experience, and 95.7% considered working in a cross-cultural team an enrichment. A total of 68.5% of respondents did not find it an imposition if people living in Israel cannot speak Hebrew properly, and 85.7% found it important to treat clients according to their cultural needs and individual values. Finally, 61.4% expressed a willingness to receive training in working with clients from different cultures.

However, as our findings indicate, general openness and awareness do not always translate into effective practical competence—especially when working with culturally and ideologically distant groups such as the Haredim. The scores on the *skills* scale indicate a strong awareness of cultural factors in healthcare, with most therapists acknowledging the importance of cultural factors in healthcare and being willing to adapt their communication strategies accordingly. Only 10% of respondents agreed that the disease concepts of clients from different cultural backgrounds are irrelevant to treatment success, and 74.3% stated that it takes more time to explain the treatment options to clients who do not understand spoken Hebrew well. Most STs (82.8%) agreed that the culturally specific factors of people (e.g., values, behavioral norms, and beliefs) influence their understanding of disease significantly and should therefore be assessed and taken into consideration by healthcare professionals.

When looking at the scores of the knowledge and attitudes toward working with the UO sector (Table 3), we divided the questions into two categories: *views* (12 items: 7, 8, 9, 10, 14, 15, 19, 20, 21, 31, 32) and *awareness* (13 items: 1, 4, 6, 12, 13, 17, 23, 24, 25, 26, 27, 28, 29) (for the full results, see Supplementary Material S3, Table S2).

The scores for the *views* scale reveal a complex and sometimes contradictory view: STs struggle with cultural gaps and conflicts but also grow professionally and gain new perspectives. Roughly the same number of respondents agreed (40%) and disagreed (37.1%) that working with UO clients is no different than working with secular clients. A total of 44.2% of respondents agreed that regardless of their lifestyle, Haredi and secular clients are basically the same. A total of 61.4% disagreed that they have to be extra careful with how they speak with their UO clients, which affects the flow of the sessions, and half (50%) of them agreed that it takes them longer to achieve trust. A total of 28.6% agreed that working with UO clients is harder for them than treating secular clients, and only 27.2% of respondents said that they enjoy working with UO clients. A total of 28.6% of respondents felt that the Haredim do not respect the secular majority, and 44.3% found differences in this sector between how they present themselves externally and what they really think, but the high result of 71.5% of respondents said they feel that their Haredi clients have changed their opinion on seculars in general, after being in therapy with them, and close to half (48.6%) disagreed that they were surprised that a UO client chose a secular therapist.

The scores for the *awareness* scale also highlight a mixed and nuanced picture among STs. While some struggled with cultural differences and trust building, others recognized unexpected similarities and insights. While only 37.2% of respondents would recommend other STs to work with UO clients, 57.2% agreed that working with UO clients has made them better therapists. Additionally, 25.7% felt that working with UO clients has changed them as a person. While roughly the same number of respondents agreed (32.8%) and disagreed (35.7%) that working with the UO sector raised professional conflicts in their work as therapists, 41.5% felt that they had to leave their personal opinions out of the room to succeed in treating UO clients. Nearly half of STs felt offended by things their UO clients said regarding secular individuals in general (45.7%), and many disagreed with having learned some aspects of UO life that are preferable to secular norms (47.2%). On the other hand, 52.9% were surprised by what they did not know about the UO community, even though 45.8% disagreed that the UO community is much different than what they first thought. Interestingly, 67.2% of STs declared they had changed their opinion on the UO sector after working with Haredi clients.

It is worth noting that respondents’ answers were more diverse and polarized when referring specifically to the UO sector compared to when they answered general questions about minorities. This suggests that therapists hold more consistent and possibly more neutral views when thinking about minority groups broadly, whereas their attitudes, perceptions, and experiences with the Haredi population tend to be more complex, emotionally charged, or uncertain. These differences likely reflect the unique cultural, religious, and ideological tensions between secular and Haredi communities in Israel, which influence how therapists perceive and engage with UO clients in particular.

The statistical analysis of the correlation between these two questionnaires (Table 4) revealed positive correlations between the various scales of the CCCHP-27 questionnaire. Higher scores on one scale were associated with higher scores on another: positive emotions correlated with positive attitudes (ρ = 0.310, *p* = 0.009), and positive attitudes correlated with higher skills (ρ = 0.268, *p* = 0.025). Similarly, a positive correlation was found between the two scales assessing competencies in working with Haredi clients: positive views were associated with higher awareness (ρ = 0.369, *p* = 0.002).

However, a negative correlation was observed between the emotions about minorities scale and both scales related to working with the UO sector—awareness of the UO sector (ρ = −0.534, *p* < 0.001) and views (ρ = −0.307, *p* = 0.01). This suggests that therapists who report strong positive emotions toward minorities, in general, may have lower cultural awareness and hold less neutral or informed views regarding the UO community.

The statistical analysis of the cross-cultural competencies according to sociodemographic characteristics (Table 5) indicates that gender differences do not significantly affect competencies or attitudes, so as professional experience and experience specifically working with Haredi clients, and the age group of clients. A factor significant to the results was found in the profession. Psychologists report significantly lower emotional responses (M = 3.53, *p* < 0.05) compared to art therapists (M = 3.85) and social workers (M = 3.90). The number of Haredi clients affects their views: therapists who have worked with more than 20 UO clients scored significantly higher on views (M = 3.30, *p* < 0.05) than those with fewer clients (M = 2.95). The workplace affects awareness: therapists who only work in public settings scored significantly lower on awareness (M = 2.48) compared to those in private settings (M = 2.95, *p* < 0.05) or both public and private settings (M = 2.71).

## 4. Discussion

The results of this study indicate two important findings. Firstly, it shows that there is a significant difference in STs’ declared attitudes toward cultural minorities versus the Haredi population. Secondly, this study demonstrates that most STs have expressed openness and willingness to work with the Haredim. While STs show a relatively high level of cultural competence, Schuster, Elroy, and Rosen reported in 2018 that, on average, the cultural competence of Israeli hospitals is “low to moderate” in terms of the local Ministry of Health and international standards [31]. In another study examining the cross-cultural competence of mental health nurses in Israel, it was found that they perceived themselves as having a moderate level of cultural competence [32].

However, in comparison to their high scores, the participants’ attitudes toward UO clients are more complex and polarized, with a low level of cultural competence. A negative correlation between all scales was also found in a Finnish study using the CCCHP-27 questionnaire, with clients who were perceived as “difficult” and who had strong criticisms of the health care provider [33]. These two cross-cultural positions reflect Israel’s complex arena in which the STs work. Their integrity and professionalism require them to present adequate therapy to all, demonstrating equality of opportunity in healthcare and a self-reflection on their views and biases [4,34], but these guidelines are put to the test when clients belong to a group that challenges the core of the profession and the interface of the delicate fabric making up Israel’s social structure [15].

On the professional level, the divide between Western and religious worldviews lies in their priorities: Western culture values individual freedom and autonomy, while religious traditions emphasize obedience, community commitment, and conformity. Individualistic cultures promote self-expression and personal agency, while collectivist cultures prioritize group identity, tradition, and social cohesion. Despite the growing awareness of multiculturalism, psychotherapy remains rooted in Western individualism, often overlooking collectivist values and even demonstrating antagonism toward the recognition of religion as pertinent to the individual [35], leading to tensions between psychotherapy and religious communities [19], and causing researchers of psychology to ignore the influences of religion on therapy [36], even though spiritual or religious adaptations to psychotherapy effectively benefit clients [37].

A prevalent belief in Judaism is that the challenges faced by Jewish individuals are divine tests that faithful Jews are expected to overcome without external assistance [38]. And, as the UO sector makes such great efforts to minimize communication with other sectors in order to avoid external influences [20], in the USA, many Haredi seeking therapy may prefer a non-Jewish therapist who understands and respects Judaism over a secular Jewish therapist who might impose non-religious corruptive perspectives in the treatment [39]. Thus, STs may feel that their professional authority is being questioned.

On the national level, the divide between STs and UO clients lies in their sharply opposing views on various public policy issues, such as marriage, divorce, military service, gender segregation, and public transportation. The UO Jews believe that Israel’s government should uphold and promote religious values, whereas secular Jews strongly advocate for the separation of religion and state [40]. These opposing views have direct influences on many aspects of Israelis’ communal existence, raising harsh negative sentiments that have reached new peaks of struggle regarding military enlistment for UO Jews since the beginning of the war in October 2023, when the secular Jews felt burdened carrying all the weight of the war, and the UO Jews fiercely resisted enlisting [41].

These findings reflect the second phase of a broader research project, designed from the outset as a two-stage study. While the first qualitative phase provided in-depth insights into therapists’ subjective experiences, the current quantitative phase tests whether these challenges—such as ideological distance, outsider status, or value conflict—can be observed and measured at scale. This structure allows for the validation and expansion of the initial insights through broader generalization.

Our study’s findings reflect this extreme social–political arena, demonstrating a gap between STs views on cross-culture therapy vs. therapy with Haredi clients. When respondents felt that the Haredim did not respect seculars, and the striking number of 45.7% found themselves offended as a secular person by things their ultraorthodox clients said regarding secular individuals in general; this gap is not baseless. STs’ professional vs. personal positions were also discussed by Be’eri et al. [42] when new dress code instructions, implemented in a clinical center to accommodate the stricter modesty standards of UO patients, created a sense of alienation among STs, who perceived these rules as an imposed cultural constraint. Those unable to align with the new institutional policy were not permitted to continue working. These findings are in line with a previous study showing that STs working with Haredi clients often experience a feeling of being an “outsider”. Moreover, they also experienced intense emotional and ideological conflicts, as deep societal divisions in Israel evoke frustration, moral discomfort, and professional strain in the therapeutic relationship. Even though many described personal growth and increasing empathy through therapeutic work, they also stressed colliding viewpoints and struggled with distrust and ideological distance (3). These patterns, previously described qualitatively, are now operationalized and statistically analyzed, offering structured insights into the prevalence and correlations between therapists’ experience, competence, and perception gaps.

Despite differences in sectors or backgrounds, the respondents expressed a strong sense of unity and shared human experiences. They emphasized that, at the core, all people are fundamentally the same, facing similar challenges, concerns, and needs. This perspective suggests that the fundamental issues people deal with are universally relatable. This shared understanding fosters a sense of cohesion, empathy, and the belief that collaboration and collective problem-solving can transcend sectoral boundaries. This search for commonality was discussed in other studies [43].

These findings reflect the two approaches therapists adopt when working with culturally different clients [19]: The Distancing Approach—therapists using this approach emphasize cultural differences and are highly aware of value conflicts. While they can clearly identify cultural significance, this focus may lead to gaps resulting in frustration or tension. The Bring Closer Approach—these therapists aim to set aside their personal values and fully embrace the client’s culture, experiencing fewer conflicts. However, this form of “color-blindness”, while good-intentioned, can sometimes blur important cultural differences, making it harder to fully understand the client’s experience [19,44].

Scholars of cross-cultural therapy have stressed the responsibility that psychotherapists have to recognize differences as social constructs that shape power dynamics and their roles within them, and to observe the injustice so as to not unconsciously replicate social order in the therapy room, potentially reactivating our patients’ socially induced negative experiences [45,46]. Our study contributes to this literature by examining a context in which therapists themselves—rather than clients—experience injustice or marginalization within the therapy setting, a dynamic underexplored in current research [3].

Our study presents findings that reflect the unique situation in Israel when the therapist is the one who experiences injustice, and, thus, the gaps in the two sets of competencies. But, as the therapist holds a role to strive for social change [45], the findings also present their willingness to receive training, which improves cross-cultural competencies, as was found in other studies using CCCHP-27 [33,47], and the respondents’ open-mindedness and awareness, as more than half said that they are often surprised by what they do not know about the ultraorthodox community, and most respondents said that many times, they are surprised by what their ultraorthodox clients do not know about the secular community. Importantly, a previous study by Doron et al. [3] also revealed that many STs feel they undergo a personal transformation through their work with Haredi clients, gaining humility, deeper cultural sensitivity, and a renewed appreciation for diverse worldviews. These findings contextualize our results by underscoring the dual nature of intercultural therapy as both challenging and enriching.

Perhaps one of the most important aspects of this encounter is that most respondents felt that both sides, them and their clients, have changed their opinions following the therapeutic interactions, which offers an explanation for the higher score on the *views* scale by those who had a larger number of Haredi clients, offering more opportunities for change.

## 5. Limitations

Although, to the best of our knowledge, this is the first study to assess cross-cultural competencies and attitudes towards ultraorthodox clients among Israeli STs, it has some limitations that should be acknowledged when interpreting the findings. Firstly, although it was designed as a pilot study, the sample size was small, with only 70 secular therapists completing the survey. Consequently, while the results cannot be extrapolated for the entire population of STs in Israel, it would be desirable to compare the findings to a larger group. While broader patterns in Israel’s healthcare and educational systems—which are predominantly secular in nature—suggest that the majority of certified STs in Israel are trained and practice within a secular framework, we fully acknowledge that without official figures, it is not possible to determine the total number of secular STs or the proportion our sample represents. It should, nevertheless, be stressed that since there is no formal registry of STs dealing with UO clients in Israel, the exact number of such therapists is unknown. Moreover, the inclusion criteria (at least three years of therapeutic experience with Haredi and secular clients and having at least two UO clients) have further limited the number of possible respondents. Furthermore, according to the recently adopted regulation, only Haredi therapists will be able to provide therapy to UO clients. More importantly, since this study was conducted in the middle of the current Israeli–Gaza war, it severely hindered the recruitment process, as many eligible participants were either unable to participate or unwilling to do so due to higher levels of stress. Due to all these reasons, the survey’s findings are limited to the perspectives of STs who consented to participate in the research and cannot be generalized to all therapists in Israel.

Non-random sampling was another limitation, as it made it impossible to analyze the sociodemographic, structural, and socio-cultural context of the topics covered in the study. Thirdly, due to the small number of participants, neither the CCCHP tool developed by Bernhard et al., [27] nor the original questionnaire designed for this study was validated. Consequently, measurement errors may have occurred, and caution should be taken when interpreting the findings. Fourthly, there is a possibility of implicit gender bias because most study participants were women. However, it should be stressed that this gender disparity reflects the dominance of women in Israel’s therapeutic fields. Fifthly, since, due to the current political and social situation in Israel, this study was conducted as a computer-assisted online survey and participants were recruited via social media and groups for the STs on FB and WhatsApp, there is a risk of recruitment bias, as some STs may not have been invited to participate or do not feel comfortable using electronic devices. Finally, the self-reported and subjective character of the survey also limits this study.

Moreover, the exploratory nature of the research and the use of non-probabilistic sampling restrict the ability to generalize the findings. While the structured design and operationalized constructs increase analytical rigor, the study still relies on voluntary participation and subjective assessments, which may introduce self-selection and social desirability bias. Future research should aim to replicate these findings using larger, more representative samples across diverse clinical settings.

It should also be noted that this research was conceived as part of a broader, two-phase study, with the present quantitative stage building directly upon an earlier qualitative investigation. While this design strengthens the study’s theoretical grounding, it also creates a degree of interdependence between phases. As such, some analytical categories and conceptual assumptions used here stem from the qualitative phase, potentially shaping the framing of questions and interpretation of results

Despite these limitations, this study has certain advantages. Most importantly, given the absence of prior research, this research sheds light on the unprecedented complexities of cross-cultural therapy with Haredi clients and fills a knowledge gap about Israeli’ STs’ cross-cultural competencies. This study may stimulate further discussion on the need for therapists working with UO clients to improve their cross-cultural competence.

## 6. Conclusions

The study highlights gaps in cross-cultural therapy shaped by Israel’s societal divides and secular–UO conflicts, influencing STs’ perceptions. Despite challenges, many therapists grow professionally through UO–client interactions. The key insights emphasize the need for training to equip therapists working with diverse populations, particularly the Haredi sector, and the importance of direct exposure for fostering cultural understanding. This need is especially urgent given the recent trends in Israeli public mental health services that increasingly assign Haredi therapists to Haredi clients, thereby limiting opportunities for cross-cultural familiarity and dialogue.

Based on the study’s findings, we recommend the following:

1. Culturally tailored training—programs should directly address the religious, social, and normative characteristics of the Haredi community rather than relying solely on generalized models of cross-cultural competence.
2. Addressing personal biases—therapists should be encouraged to explore and reflect on their own emotions, assumptions, and potential biases toward religious populations, especially the Haredim.
3. Including community perspectives—educational initiatives should involve voices from different local communities, including the Haredi community—such as therapists or community leaders—to bridge knowledge gaps and counteract stereotypes.
4. Re-examining structural barriers—the current organization of mental health services (e.g., same-community therapist-client matching) should be reconsidered, as it may inadvertently reduce opportunities for meaningful cross-cultural engagement and learning.

At the same time, we believe that future research should examine the long-term impacts of culturally tailored training on therapists’ competencies and attitudes, especially toward minority populations like the Haredim. Comparative studies across cultural or religious groups, within Israel and in other multicultural settings, could also enhance the generalizability of these findings. Additionally, qualitative research into the lived experiences of both therapists and Haredi clients would offer deeper insights into the mutual perceptions and therapeutic dynamics. Future studies should also consider method triangulation, such as comparing the perspectives of secular therapists and Haredi clients directly, to better understand the therapeutic dynamics. Evaluating the effectiveness of specific training programs over time and conducting longitudinal research to assess whether sustained contact with UO clients leads to enduring changes in therapists’ attitudes would also be valuable. Finally, exploring how institutional policies shape cross-cultural interactions could support the development of more inclusive mental health services.

## Figures and Tables

**Table 1:** Sociodemographic characteristics of secular therapists.

Characteristics	Total	
	N = 70	%
Sex		
female	61	87.1
male	9	12.9
Profession		
psychologist	26	37.1
art therapist	23	32.9
clinical social worker	21	30
Age (in years)		
range	33–74	
mean (95% CI)	49 (46.7–51.3)	
SD	9.7	
median	48	
Professional experience (in years)		
range	4–50	
mean (95% CI)	18.3 (16.1–20.4)	
SD	9	
median	16	
Professional experience working with Haredi clients (in years)		
range	2–44	
mean (95% CI)	11.3 (9.1–13.5)	
SD	9.2	
median	9	
Number of Haredi clients		
range	2–300	
mean (95% CI)	29 (17–41)	
SD	50.1	
median	10	
Place of work		
public	27	38.6
private	10	14.3
public and private	33	47.1
Age group of clients		
children	14	20
adults	20	28.6
both	36	51.4

**Table 2:** Secular therapists’ cross-cultural competencies (CCCHP-27).

Subscale	Question	Completely Agree	Mostly Agree	Neither Agree nor Disagree	Mostly Disagree	Completely Disagree
	I often find it difficult to relate to the elaborations of my clients when their socio-cultural background is quite different from mine.	3 (4.3)	15 (21.4)	20 (28.6)	26 (37.1)	6 (8.6)
	Being part of a different cultural group is a critical life experience and can be accompanied by psychosocial stress and health burdens.	18 (25.7)	29 (41.4)	17 (24.3)	5 (7.1)	1 (1.4)
*Emotions*	I find it challenging to speak slowly and use clear, straightforward language with people who have difficulty understanding my instructions.	1 (1.4)	4 (5.7)	12 (17.1)	16 (22.9)	37 (52.9)
	I prefer treating clients from my cultural background to those who seem foreign.	1 (1.4)	11 (15.7)	20 (28.6)	21 (30)	17 (24.3)
	In my professional interaction with clients from different cultural backgrounds, I often feel unsure, angry, and frustrated.	0	9 (12.9)	20 (28.6)	25 (35.7)	16 (22.9)
	I get impatient when I cannot make myself understood by clients from different cultural backgrounds.	0	8 (11.4)	16 (22.9)	21 (30)	25 (35.7)
	I consider working in a cross-cultural team an enrichment.	50 (71.4)	17 (24.3)	2 (2.9)	0	1 (1.4)
	I find it an imposition when people who live in Israel cannot speak Hebrew properly.	2 (2.9)	4 (5.7)	16 (22.9)	22 (31.4)	26 (37.1)
	I enjoy talking to people of different cultural backgrounds about their experiences here.	24 (34.3)	28 (40)	15 (21.4)	1 (1.4)	2 (2.9)
*Attitudes*	The interaction with people from other cultural backgrounds helps me reflect upon my cultural background.	20 (28.6)	35 (50)	10 (14.3)	4 (5.7)	1 (1.4)
	I would like to use training, advising, and educational offers to improve my understanding of clients from different cultural backgrounds.	11 (15.7)	32 (45.7)	15 (21.4)	7 (10)	5 (7.1)
	It is important for me to treat clients according to their cultural needs and individual values.	28 (40)	32 (45.7)	10 (14.3)	0	0
	I find it exciting to treat clients from different cultural backgrounds.	18 (25.7)	19 (27.1)	31 (44.3)	1 (1.4)	1 (1.4)
	Within the different sectors of the Israeli population, there are hardly any differences in terms of health opportunities and disease risks.	2 (2.9)	6 (8.6)	19 (27.1)	23 (32.9)	20 (28.6)
	The disease concepts of clients from different cultural backgrounds are irrelevant to treatment success.	2 (2.9)	5 (7.1)	8 (11.4)	26 (37.1)	29 (41.4)
*Skills*	I take more time explaining the treatment options to clients who do not understand spoken Hebrew well.	22 (31.4)	30 (42.9)	15 (21.4)	3 (4.3)	0
	With clients who do not understand spoken Hebrew very well, I take more time to discuss their expectations and fears.	17 (24.3)	22 (31.4)	23 (32.9)	8 (11.4)	0
	The culturally specific factors of people (e.g., values, behavioral norms, and beliefs) influence their understanding of disease significantly and should therefore be assessed and taken into consideration by healthcare professionals.	29 (41.4)	29 (41.4)	11 (15.7)	1 (1.4)	0

**Table 3:** Secular therapists’ attitudes toward the ultraorthodox sector.

Subscale	Question	Completely Agree	Mostly Agree	Neither Agree nor Disagree	Mostly Disagree	Completely Disagree
*Views*	Working with ultraorthodox clients is harder for me than treating secular clients.	4 (5.7)	16 (22.9)	19 (27.1)	16 (22.9)	15 (21.4)
	I find working with ultraorthodox clients no different than working with secular clients.	8 (11.4)	20 (28.6)	16 (22.9)	18 (25.7)	8 (11.4)
	Regardless of their lifestyle, ultraorthodox and secular clients are basically the same.	12 (17.1)	19 (27.1)	30 (42.9)	6 (8.6)	3 (4.3)
	I feel that my ultraorthodox clients have changed their opinion on secular individuals after being in therapy with me.	16 (22.9)	34 (48.6)	17 (24.3)	1 (1.4)	2 (2.9)
	I enjoy working with ultraorthodox clients.	6 (8.6)	13 (18.6)	16 (22.9)	17 (24.3)	18 (25.7)
	Ultraorthodox clients seek help for the same reasons secular clients turn to therapy.	3 (4.3)	27 (38.6)	27 (38.6)	13 (18.6)	19 (27.1)
	I feel that ultraorthodox clients don’t respect secular individuals.	4 (5.7)	16 (22.9)	23 (32.9)	19 (27.1)	8 (11.4)
	I have to be extra careful with how I speak with my ultraorthodox clients, which affects the flow of the sessions.	1 (1.4)	9 (12.9)	17 (24.3)	32 (45.7)	11 (15.7)
	I put a lot of energy into identifying my biases toward the ultraorthodox sector.	0	17 (24.3)	19 (27.1)	25 (35.7)	9 (12.9)
	I often find differences in this sector between how they present themselves externally and what they really think.	3 (4.3)	28 (40)	24 (34.3)	12 (17.1)	3 (4.3)
	I often feel provoked by my ultraorthodox clients.	0	6 (8.6)	13 (18.6)	20 (28.6)	31 (44.3)
	It takes longer to achieve trust in therapy with ultraorthodox clients.	10 (14.3)	25 (35.7)	16 (22.9)	12 (17.1)	7 (10)
	I was surprised that an ultraorthodox client chose a secular therapist.	2 (2.9)	15 (21.4)	19 (27.1)	20 (28.6)	14 (20)
*A* *wareness*	I would recommend other secular therapists to work with ultraorthodox clients.	6 (8.6)	20 (28.6)	11 (15.7)	19 (27.1)	14 (20)
	During my work, I learned some aspects of ultraorthodox life that are preferable to secular norms.	6 (8.6)	13 (18.6)	18 (25.7)	20 (28.6)	13 (18.6)
	Working with the ultraorthodox sector raises professional conflicts in my work as a therapist.	5 (7.1)	18 (25.7)	22 (31.4)	14 (20)	11 (15.7)
	After working with ultraorthodox clients, I have changed my opinion on the ultraorthodox sector.	17 (24.3)	30 (42.9)	11 (15.7)	9 (12.9)	3 (4.3)
	I found myself offended as a secular person by things my ultraorthodox clients said regarding secular individuals in general.	7 (10)	25 (35.7)	24 (34.3)	10 (14.3)	4 (5.7)
	I am often surprised by what I don’t know about the ultraorthodox community.	10 (14.3)	27 (38.6)	24 (34.3)	7 (10)	2 (2.9)
	I have to leave my personal opinions out of the room to succeed in treating ultraorthodox clients.	2 (2.9)	27 (38.6)	19 (27.1)	15 (21.4)	7 (10)
	The ultraorthodox community is much different than what I first thought.	2 (2.9)	14 (20)	22 (31.4)	23 (32.9)	9 (12.9)
	Working with ultraorthodox clients has made me a better therapist.	10 (14.3)	30 (42.9)	18 (25.7)	7 (10)	5 (7.1)
	I always feel like there are three of us in the room—me, the client, and the rabbi.	1 (1.4)	15 (21.4)	16 (22.9)	21 (30)	17 (24.3)
	Working with ultraorthodox clients has changed me as a person.	3 (4.3)	15 (21.4)	21 (30)	17 (24.3)	14 (20)

**Table 4:** Correlations between secular therapists’ cross-cultural competencies and their attitudes toward the ultraorthodox sector.

	Spearman’s Rho	95% CI	*p*
Factor 1 (Emotions)—Factor 2 (Attitudes)	0.310	0.074; 0.531	**0.009**
Factor 1 (Emotions)—Factor 3 (Skills)	0.114	−0.113;0.300	ns
Factor 1 (Emotions)—Index S1 (Views)	−0.307	−0.508; −0.083	**0.01**
Factor 1 (Emotions)—Index S2 (Awareness)	−0.534	−0.698; −0.279	**<0.001**
Factor 2 (Attitudes)—Factor 3 (Skills)	0.268	0.071; 0.458	**0.025**
Factor 2 (Attitudes)—Index S1 (Views)	0.224	−0.054; 0.457	ns
Factor 2 (Attitudes)—Index S2 (Awareness)	−0.067	−0.342; 0.163	ns
Factor 3 (Skills)—Index S1 (Views)	0.185	−0.072; 0415	ns
Factor 3 (Skills)—Index S2 (Awareness)	0.091	−0.172; 0.331	ns
Index S1 (Views)—Index S2 (Awareness)	0.369	0.115; 0.583	**0.002**

Statistically significant differences are in bold; ns: not significant.

**Table 5:** Secular therapists’ cross-cultural competencies and attitudes toward the ultraorthodox sector by sociodemographic characteristics.

	Factor 1 Emotions	Factor 2 Attitudes	Factor 3 Skills	Index S1 Views	Index S2 Awareness
Sex					
female (*n* = 61)	3.74	4.01	3.98	3.05	2.66
male (*n* = 9)	3.80	4.06	3.78	3.21	2.61
p	ns	ns	ns	ns	ns
Profession					
psychologist (*n* = 26)	3.53	3.96	4.07	3.18	2.84
art therapist (*n* = 23)	3.85	3.99	3.91	2.85	2.51
social worker (*n* = 21)	3.90	4.10	3.85	3.18	2.59
p	**<0.05**	ns	ns	ns	ns
Professional experience					
up to 14 years (*n* = 21)	3.77	3.99	4.00	3.11	2.64
over 15 years (*n* = 49)	3.73	4.02	3.93	3.06	2.66
p	ns	ns	ns	ns	ns
Professional experience working with Haredi clients					
up to 9 years (*n* = 36)	3.74	4.13	3.98	3.14	3.66
over 10 years (*n* = 34)	3.75	3.90	3.92	2.99	3.65
p	ns	ns	ns	ns	ns
Number of Haredi clients					
up to 19 (*n* = 45)	3.73	3.99	3.92	2.95	2.65
over 20 (*n* = 25)	3.77	4.06	4.02	3.30	2.67
p	ns	ns	ns	**<0.05**	ns
Place of work					
public (*n* = 27)	3.77	4.07	3.84	3.14	2.48
private (*n* = 10)	3.63	4.01	3.94	2.93	2.95
public and private (*n* = 33)	3.76	3.97	4.04	3.06	2.71
p	ns	ns	ns	ns	**<0.05**
Age group of clients					
children (*n* = 14)	3.74	3.86	3.89	2.72	2.50
adults (*n* = 20)	3.81	4.15	3.88	3.30	2.64
both (*n* = 36)	3.71	4.00	4.02	3.08	2.73
p	ns	ns	ns	ns	ns

Statistically significant differences are in bold; ns: not significant.

## Data Availability

The data supporting this study’s findings are not openly available due to reasons of sensitivity and are available from the corresponding author upon reasonable request.

## Supplementary Materials

The following supporting information can be downloaded at https://www.mdpi.com/article/10.3390/healthcare13101210/s1. Supplementary Material S1. Online Questionnaire; Supplementary Material S2. Results of Secular therapists’ cross-cultural competencies CCCHP-27 questionnaire; Supplementary Material S3. Results of Secular therapists’ knowledge and attitudes towards working with the UO sector questionnaire.

## Abbreviations

CCCHP: Cross-Cultural Competence of Healthcare Professionals; STs: secular therapists; UO: ultraorthodox.
