# Supplementary material for: The Cross-Cultural Competencies and Attitudes Toward Ultraorthodox Clients Among Secular Therapists in Israel: An Explanatory Study
Source: Healthcare (Basel). 2025 May 21;13(10):1210. doi: 10.3390/healthcare13101210 (PMC12111204; doi:10.3390/healthcare13101210)
Supplement: Supplementary file 1 [file healthcare-13-01210-s001.zip › healthcare-3581730-supplementary/Supplementary material 1.pdf]

Supplementary material S1

Online questionnaire

Dear Madam! Dear Sir!
Thank you for your willingness to participate in the research I am conducting as part of my doctoral dissertation at Poznan University of Medical Sciences, under the supervision of Prof. Domaradzki from the Faculty of Social Sciences.
This study aims to explore your perspectives and experiences as a secular therapist working with minorities and various sectors in Israel. The study has been approved by the Bioethics Committee, approval number KB-139/23.
At the end of the questionnaire, you will find my contact details should you wish to get in touch.
The survey consists of three parts:

1. **Demographic information**
2. **32 questions** regarding your attitudes towards therapy with minority and diverse populations. This section is based on a validated questionnaire, which we have received written permission to use from Dr. Bernhard of the Department of Medical Psychology at the University of Hamburg.
3. 32 questions regarding your attitudes towards therapy with a specific sector.

The survey is anonymous, and respondents cannot be identified.
Thank you very much for your willingness to help and participate!
Your feedback is very important. I appreciate your time and help.

Before you start, please fill in the following declaration:

Consent Form

I, the undersigned, agree to participate in the research project titled *Experiences of Secular Therapists with Intercultural Therapy Involving Ultra-Orthodox Jews in Israel, conducted by Ms Einat Doron,* who has discussed the research project with me.

I have received and read a copy of the information letter and have had the opportunity to ask questions about this research and received satisfactory answers.

I hereby give consent to participate in the research project, and the following has been explained to me:

- the purpose and methods of this study
- the research will not benefit me
- my participation is entirely voluntary
- my right to withdraw from the study at any time without any implications to me
- what I am expected to do
- whom I can contact for any queries regarding the study
- that no personally identifiable information is captured
- all information gathered will be used only for scientific purposes.

I also consent to:

- publication of results from this study.

yes no

There are three eligibility criteria for participating in the study.
Please mark in the designated boxes to confirm that you meet all of them.

☐ I am secular (not traditional and not formerly religious).
☐ I have over two years of experience in the profession.
☐ I have had at least two Haredi (ultra-Orthodox) clients (not formerly secular, not from the Religious Zionist community, and not supervisees).

Part 1. In this part, I ask about your demographic characteristics

Profession
…………………………………

Age
…………………………………..

Sex
Woman
Man
I prefer not to say

Years of professional experience
…………………………………..

Years of professional experience working with haredim
………………………………….

Age group of clients
children
adults
both

Type of practice
private
public
private and public

Overall number of Haredi clients over the years
…………………………………

Part 2. This section consists of 32 questions. You are asked to select the statement that best describes your feelings about working with Haredi clients.

1 = I strongly disagree
2 = I disagree
3 = I neither agree nor disagree
4 = I agree
5 = I strongly agree

After each statement, please only mark the box for the most applicable answer.	Completely agree	Mostly agree	Neither agree nor disagree	Mostly disagree	Completely disagree	No answer possible
1. Working with ultraorthodox clients is harder for me than treating seculars.						
2. I believe that every therapist should work inter-culturally.						
3. Every ultraorthodox client benefits from treatment with a secular therapist.						
4. I find working with ultraorthodox clients no different from working with secular clients.						
5. I feel I should have had more training specific to the ultraorthodox community before working with ultraorthodox clients.						
6. Regardless of their lifestyle, ultraorthodox and secular clients are basically the same.						
7. I would recommend other secular therapists to work with ultraorthodox clients.						
8. During my work, I learned some aspects of ultraorthodox life that are preferable to the secular norms.						
9. Working with the ultraorthodox sector raises professional conflicts in my work as a therapist.						
10. After working with ultraorthodox clients, I have changed my opinion on the ultraorthodox sector.						
11. I feel that both sectors are highly misunderstood by one another.						
12. I feel that my ultraorthodox clients have changed their opinion on seculars after being in therapy with me.						
13. I enjoy working with ultraorthodox clients.						
14. I found myself offended as a secular person by things my ultraorthodox clients said regarding seculars in general.						
15. I am often surprised by what I don’t know about the ultraorthodox community.						
16. Many times, I am surprised by what my ultraorthodox clients don’t know about the secular community.						
17. Ultraorthodox clients seek help for the same reasons seculars turn to therapy.						
18. Ultraorthodox clients wouldn’t achieve what they did, going to an ultraorthodox therapist.						
19. I have to leave my personal opinions out of the room to succeed in treating ultraorthodox clients.						

20. The ultraorthodox community is much different than what I first thought.						
21. Working with ultraorthodox clients has made me a better therapist.						
22. There are many more common things than differentiating between seculars and ultraorthodox.						
23. I feel that ultraorthodox don't respect seculars						
24. I have to be extra careful with how I speak with my ultraorthodox clients, which affects the flow of the sessions.						
25. I put a lot of energy into identifying my biases towards the ultraorthodox sector.						
26. I often find differences in this sector between how they present themselves externally and what they really think.						
27. I often feel provoked by my ultraorthodox clients.						
28. It takes longer to achieve trust in therapy with ultraorthodox clients.						
29. I was surprised that an ultraorthodox client chose a secular therapist.						
30. Ultraorthodox people are stronger-minded than I thought and don't necessarily follow what they are told.						
31. I always feel like there are three of us in the room-me, the client, and the Rabbi.						
32. Working with ultraorthodox has changed me as a person.						

Part 3. This section consists of 32 questions in which you are asked to select the statement that best describes your feelings about working with clients from minority groups and different sectors in Israel.

After each statement, please only mark the box for the most applicable answer.	Completely agree	Mostly agree	Neither agree nor disagree	Mostly disagree	Completely disagree	No answer possible
1. I consider working in a cross-cultural team an enrichment.						
2. To achieve the agreed treatment goal, I ask clients from different cultural backgrounds what they need regarding support.						
3. I find it an imposition when people who live in Israel cannot speak Hebrew properly.						
4. Within the different sectors of the Israeli population, there are hardly any differences in terms of health opportunities and disease risks.						
5. By communicating with clients from different cultural backgrounds, I can learn about different life orientations.						
6. My professional perception, assessment, and behavior remain untouched by my cultural imprinting.						

7. Cultural diversity is also an enrichment.						
8. I enjoy talking to people of different cultural backgrounds about their experiences here.						
9. I often find it difficult to relate to the elaborations of my clients when their socio-cultural background is quite different from mine.						
10. I do not differentiate between clients and treat all equally, even though it is sometimes difficult to communicate.						
11. I have the impression that people from different cultural backgrounds often assume discrimination when, in fact, general rules are simply being enforced.						
12. Being part of a different cultural group is a critical life experience and can be accompanied by psychosocial stress and health burdens.						
13. I find speaking slowly in lay language challenging with people who struggle to understand my instructions.						
14. I always remain friendly and courteous with people from different cultural backgrounds, even when stressed out.						
15. The interaction with people from other cultural backgrounds helps me reflect upon my cultural background.						
16. The disease concepts of clients from different cultural backgrounds are irrelevant to treatment success.						
17. In a conversation, I always listen attentively and let individuals from different cultural backgrounds finish their sentences.						
18. During arguments with people from different cultural backgrounds, I always remain factual and objective.						
19. I would like to use training, advising, and educational offers to improve my understanding of clients from different cultural backgrounds.						
20. I consider it an enrichment to have friendships with people from different cultural backgrounds.						
21. People living in Israel should adapt to the general society, not the other way around.						
22. I take more time explaining the treatment options to clients who do not understand spoken Hebrew well.						
23. I prefer treating clients from my cultural background to those who seem foreign.						
24. With clients who do not understand spoken Hebrew very well, I take more time to discuss their expectations and fears.						
25. Culturally specific factors of people (e.g., values, behavior norms, beliefs) influence their understanding of disease significantly and should therefore be assessed and taken into consideration by healthcare professionals.						
26. I consider clients' values concerning family, religion, etc., if they seem relevant to the treatment.						

27. In my professional interaction with clients from different cultural backgrounds, I often feel unsure, angry, and frustrated.						
28. It is important for me to treat clients according to their cultural needs and individual values.						
29. Institutions and the public pay too much attention to the unique wishes of groups from different cultural backgrounds.						
30. I never hesitate to help someone with a different cultural background in case of an emergency.						
31. I get impatient when I cannot make myself understood by clients from different cultural backgrounds.						
32. I find it exciting to treat clients from different cultural backgrounds.						
