# Supplementary material for: The Cross-Cultural Competencies and Attitudes Toward Ultraorthodox Clients Among Secular Therapists in Israel: An Explanatory Study
Source: Healthcare (Basel). 2025 May 21;13(10):1210. doi: 10.3390/healthcare13101210 (PMC12111204; doi:10.3390/healthcare13101210)
Supplement: Supplementary file 1 [file healthcare-13-01210-s001.zip › healthcare-3581730-supplementary/Supplementary material 2.pdf]

Supplementary material S2

Results of Secular therapists' cross-cultural competencies CCCHP-27 questionnaire

	Completely agree	Mostly agree	Neither agree nor disagree	Mostly disagree	Completely disagree
1. I consider working in a cross-cultural team an enrichment.	50 (71.4)	17 (24.3)	2 (2.9)	0	1 (1.4)
2. To achieve the agreed treatment goal, I ask clients from different cultural backgrounds what they need regarding support.	23 (32.9)	26 (37.1)	16 (22.9)	4 (5.7)	1 (1.4)
3. I find it an imposition when people who live in Israel cannot speak Hebrew properly.	2 (2.9)	4 (5.7)	16 (22.9)	22 (31.4)	26 (37.1)
4. Within the different sectors of the Israeli population, there are hardly any differences in terms of health opportunities and disease risks.	2 (2.9)	6 (8.6)	19 (27.1)	23 (32.9)	20 (28.6)
5. By communicating with clients from different cultural backgrounds, I can learn about different life orientations.	45(64.3)	23 (32.9)	2 (2.9)	0	0
6. My professional perception, assessment, and behavior remain untouched by my cultural imprinting.	4 (5.7)	13 (18.6)	19 (27.1)	23 (32.9)	11 (15.7)
7. Cultural diversity is also an enrichment.	51 (72.9)	17 (24.3)	2 (2.9)	0	0
8. I enjoy talking to people of different cultural backgrounds about their experiences here.	24 (34.3)	28 (40)	15 (21.4)	1 (1.4)	2 (2.9)
9. I often find it difficult to relate to the elaborations of my clients when their socio-cultural background is quite different from mine.	3 (4.3)	15 (21.4)	20 (28.6)	26 (37.1)	6 (8.6)
10. I do not differentiate between clients and treat all equally, even though it is sometimes difficult to communicate.	24 (34.3)	26 (37.1)	12 (17.1)	6 (8.6)	2 (2.9)
11. I have the impression that people from different cultural backgrounds often assume discrimination when, in fact, general rules are simply being enforced.	2 (2.9)	10 (14.3)	34 (48.6)	17 (24.3)	7 (10)
12. Being part of a different cultural group is a critical life experience and can be accompanied by psychosocial stress and health burdens.	18 (25.7)	29 (41.4)	17 (24.3)	5 (7.1)	1 (1.4)
13. I find speaking slowly in lay language challenging with people who struggle to understand my instructions.	1 (1.4)	4 (5.7)	12 (17.1)	16 (22.9)	37 (52.9)
14. I always remain friendly and courteous with people from different cultural backgrounds, even when stressed out.	19 (27.1)	34 (48.6)	10 (14.3)	6 (8.6)	1 (1.4)
15.The interaction with people from other cultural backgrounds helps me reflect upon my cultural background.	20 (28.6)	35 (50)	10 (14.3)	4 (5.7)	1 (1.4)
16. The disease concepts of clients from different cultural backgrounds are irrelevant to treatment success.	2 (2.9)	5 (7.1)	8 (11.4)	26 (37.1)	29 (41.4)
17. In a conversation, I always listen attentively and let individuals from different cultural backgrounds finish their sentences.	34 (48.6)	24 (34.3)	9 (12.9)	3 (4.3)	0
18. During arguments with people from different cultural backgrounds, I always remain factual and objective.	3 (4.3)	10 (14.3)	36 (51.4)	18 (25.7)	3 (4.3)

19. I would like to use training, advising, and educational offers to improve my understanding of clients from different cultural backgrounds.	11 (15.7)	32 (45.7)	15 (21.4)	7 (10)	5 (7.1)
20. I consider it an enrichment to have friendships with people from different cultural backgrounds.	34 (48.6)	29 (41.4)	7 (10)	0	0
21. People living in Israel should adapt to the general society, not the other way around.	0	5 (7.1)	36 (51.4)	21 (30)	8 (11.4)
22. I take more time explaining the treatment options to clients who do not understand spoken Hebrew well.	22 (31.4)	30 (42.9)	15 (21.4)	3 (4.3)	0
23. I prefer treating clients from my cultural background to those who seem foreign.	1 (1.4)	11 (15.7)	20 (28.6)	21 (30)	17 (24.3)
24. With clients who do not understand spoken Hebrew very well, I take more time to discuss their expectations and fears.	17 (24.3)	22 (31.4)	23 (32.9)	8 (11.4)	0
25. Culturally specific factors of people (e.g., values, behavior norms, beliefs) influence their understanding of disease significantly and should therefore be assessed and taken into consideration by healthcare professionals.	29 (41.4)	29 (41.4)	11 (15.7)	1 (1.4)	0
26. I consider clients' values concerning family, religion, etc., if they seem relevant to the treatment.	39 (55.7)	30 (42.9	1 (1.4)	0	0
27. In my professional interaction with clients from different cultural backgrounds, I often feel unsure, angry, and frustrated.	0	9 (12.9)	20 (28.6)	25 (35.7)	16 (22.9)
28. It is important for me to treat clients according to their cultural needs and individual values.	28 (40)	32 (45.7)	10 (14.3)	0	0
29. Institutions and the public pay too much attention to the unique wishes of groups from different cultural backgrounds.	0	3 (4.3)	18 (25.7)	25 (35.7)	24 (34.3)
30. I never hesitate to help someone with a different cultural background in case of an emergency.	52 (74.3)	15 (21.4)	1 (1.4)	0	2 (2.9)
31. I get impatient when I cannot make myself understood by clients from different cultural backgrounds.	0	8 (11.4)	16 (22.9)	21 (30)	25 (35.7)
32. I find it exciting to treat clients from different cultural backgrounds.	18 (25.7)	19 (27.1)	31 (44.3)	1 (1.4)	1 (1.4)
