# Supplementary material for: The Cross-Cultural Competencies and Attitudes Toward Ultraorthodox Clients Among Secular Therapists in Israel: An Explanatory Study
Source: Healthcare (Basel). 2025 May 21;13(10):1210. doi: 10.3390/healthcare13101210 (PMC12111204; doi:10.3390/healthcare13101210)
Supplement: Supplementary file 1 [file healthcare-13-01210-s001.zip › healthcare-3581730-supplementary/Supplementary material 3.pdf]

Supplementary material S3

Table S3. Results of Secular therapists' knowledge and attitudes towards working with the UO sector questionnaire

	Completely agree	Mostly agree	Neither agree nor disagree	Mostly disagree	Completely disagree	X
1. Working with ultraorthodox clients is harder for me than treating seculars.	4 (5.7)	16 (22.9)	19 (27.1)	16 (22.9)	15 (21.4)	
2. I believe that every therapist should work inter-culturally.	18 (25.7)	19 (27.1)	20 (28.6)	10 (14.3)	3 (4.3)	
3. Every ultraorthodox client benefits from treatment with a secular therapist.	4 (5.7)	12 (17.1)	32 (45.7)	17 (24.3)	5 (7.1)	
4. I find working with ultraorthodox clients no different than working with secular clients.	8 (11.4)	20 (28.6)	16 (22.9)	18 (25.7)	8 (11.4)	
5. I feel I should have had more training specific to the ultraorthodox community before working with ultraorthodox clients.	13 (18.6)	21 (30)	22 (31.4)	11 (15.7)	3 (4.3)	
6. Regardless of their lifestyle, ultraorthodox and secular clients are basically the same.	12 (17.1)	19 (27.1)	30 (42.9)	6 (8.6)	3 (4.3)	
7. I would recommend other secular therapists to work with ultraorthodox clients.	6 (8.6)	20 (28.6)	11 (15.7)	19 (27.1)	14 (20)	
8. During my work, I learned some aspects of ultraorthodox life that are preferable to secular norms.	6 (8.6)	13 (18.6)	18 (25.7)	20 (28.6)	13 (18.6)	
9. Working with the ultraorthodox sector raises professional conflicts in my work as a therapist.	5 (7.1)	18 (25.7)	22 (31.4)	14 (20)	11 (15.7)	
10. After working with ultraorthodox clients, I have changed my opinion on the ultraorthodox sector.	17 (24.3)	30 (42.9)	11 (15.7)	9 (12.9)	3 (4.3)	
11. I feel that both sectors are highly misunderstood by one another.	6 (8.6)	15 (21.4)	38 (54.3)	9 (12.9)	2 (2.9)	
12. I feel that my ultraorthodox clients have changed their opinion on seculars after being in therapy with me.	16 (22.9)	34 (48.6)	17 (24.3)	1 (1.4)	2 (2.9)	
13. I enjoy working with ultraorthodox clients.	6 (8.6)	13 (18.6)	16 (22.9)	17 (24.3)	18 (25.7)	
14. I found myself offended as a secular person by things my ultraorthodox clients said regarding seculars in general.	7 (10)	25 (35.7)	24 (34.3)	10 (14.3)	4 (5.7)	
15. I am often surprised by what I don't know about the ultraorthodox community.	10 (14.3)	27 (38.6)	24 (34.3)	7 (10)	2 (2.9)	

16. Many times, I am surprised by what my ultraorthodox clients don’t know about the secular community.	25 (35.7)	29 (41.4)	9 (12.9)	7 (10)	0	
17. Ultraorthodox clients seek help for the same reasons seculars turn to therapy.	3 (4.3)	27 (38.6)	27 (38.6)	13 (18.6)	19 (27.1)	
18. Ultraorthodox clients wouldn’t achieve what they did, going to an ultraorthodox therapist.	8 (11.4)	27 (38.6)	9 (12.9)	22 (31.4)	4 (5.7)	
19. I have to leave my personal opinions out of the room to succeed in treating ultraorthodox clients.	2 (2.9)	27 (38.6)	19 (27.1)	15 (21.4)	7 (10)	
20. The ultraorthodox community is much different than what I first thought.	2 (2.9)	14 (20)	22 (31.4)	23 (32.9)	9 (12.9)	
21. Working with ultraorthodox clients has made me a better therapist.	10 (14.3)	30 (42.9)	18 (25.7)	7 (10)	5 (7.1)	
22. There are many more common things than differentiating between seculars and ultraorthodox.	10 (14.3)	28 (40)	24 (34.3)	7 (10)	1 (1.4)	
23. I feel that ultraorthodox don’t respect seculars	4 (5.7)	16 (22.9)	23 (32.9)	19 (27.1)	8 (11.4)	
24. I have to be extra careful with how I speak with my ultraorthodox clients, which affects the flow of the sessions.	1 (1.4)	9 (12.9)	17 (24.3)	32 (45.7)	11 (15.7)	
25. I put a lot of energy into identifying my biases towards the ultraorthodox sector.	0	17 (24.3)	19 (27.1)	25 (35.7)	9 (12.9)	
26. I often find differences in this sector between how they present themselves externally and what they really think.	3 (4.3)	28 (40)	24 (34.3)	12 (17.1)	3 (4.3)	
27. I often feel provoked by my ultraorthodox clients.	0	6 (8.6)	13 (18.6)	20 (28.6)	31 (44.3)	
28. It takes longer to achieve trust in therapy with ultraorthodox clients.	10 (14.3)	25 (35.7)	16 (22.9)	12 (17.1)	7 (10)	
29. I was surprised that an ultraorthodox client chose a secular therapist.	2 (2.9)	15 (21.4)	19 (27.1)	20 (28.6)	14 (20)	
30. Ultraorthodox people are stronger-minded than I thought and don’t necessarily follow what they are told.	7 (10)	15 (21.4)	23 (32.9)	19 (17.1)	6 (8.6)	
31. I always feel like there are three of us in the room- me, the client, and the Rabbi.	1 (1.4)	15 (21.4)	16 (22.9)	21 (30)	17 (24.3)	
32. Working with ultraorthodox clients has changed me as a person.	3 (4.3)	15 (21.4)	21 (30)	17 (24.3)	14 (20)	
